# Efficacy and safety of celecoxib in schizophrenia: a systematic review and meta-analysis of randomized controlled trials

**DOI:** 10.1007/s10787-025-01908-6

**Published:** 2025-08-20

**Authors:** NourAllah Farag, Abdullah Selim, Osama Hassan, Hamdy A. Makhlouf, Mahmoud Soliman, Lamees Taman, Omar Kassar

**Affiliations:** 1 Faculty of Medicine, Suez University, Suez, Egypt; 2 Faculty of Medicine, Alexanderia University, Alexandria, Egypt; 3 Psychiatry Resident, Central and North West London NHS Foundation Trust, London, UK; 4 Faculty of Medicine, Minia University, Minia, Egypt; 5 Faculty of Medicine, Al-Azhar University, Cairo, Egypt; 6 Faculty of Medicine, Tanta University, Tanta, Egypt

**Keywords:** Celecoxib, COX-2 inhibitor, Inflammation, Meta-analysis, PANSS, Schizophrenia

## Abstract

**Background:**

The hypothesis of immune and inflammatory dysregulation has been supported in the pathogenesis of schizophrenia; this systematic review and meta-analysis aims to evaluate the efficacy and safety of celecoxib, which is a COX-2 inhibitor with anti-inflammatory effects, in the management of schizophrenia.

**Methods:**

A comprehensive search of electronic databases, including PubMed, Scopus, Cochrane, and Web of Science, was performed in February 2025. We included only randomized controlled trials (RCTs) comparing celecoxib alone or as an adjunct to antipsychotics to placebo. Risk of bias was assessed using the ROB-2 tool, and the data of included studies were extracted and analyzed using RevMan 5.4 software. The primary outcome was the change in PANSS (Positive and Negative Syndrome Scale) total score.

**Results:**

Seven RCT studies comprising 438 patients with schizophrenia were included in this study. The risk of bias was low in most of the included studies. Celecoxib showed statistically significant improvement in PANSS total score (MD = − 8.45, 95% CI [− 13.62, -3.28], *p* value = 0.001), PANSS-positive scale (MD = − 3.34, 95% CI [− 4.67, − 2.02], *P* < 0.00001), PANSS-negative sub-scales (MD = − 2.47, 95% CI [− 3.75, − 1.19], *P* = 0.00002), and general psychopathology (MD = − 4.47, 95% CI [8.10, − 0.85], *P* = 0.02). No publication bias was detected based on the DOI plot.

**Conclusion:**

This study’s findings provide insights into the potential anti-inflammatory benefits of celecoxib in schizophrenia treatment. However, study limitations warrant cautious interpretation, highlighting the need for larger, well-designed trials to identify patient subgroups that may benefit most from this approach.

**Supplementary Information:**

The online version contains supplementary material available at 10.1007/s10787-025-01908-6.

## Introduction

Schizophrenia, affecting approximately 1% of people worldwide, is characterized by positive symptoms, such as delusions and hallucinations, as well as negative symptoms including flat affect, alogia, avolition, and anhedonia (Andreasen and Olsen 1982; Velligan and Rao 2023; McCutcheon, Reis Marques, and Howes 2020). Schizophrenia has attracted many etiological theories, such as dopaminergic overactivity, glutaminergic hypoactivity, and serotonergic hyperactivity, along with emerging research about the immune and inflammatory basis of the disease (Miller and Goldsmith 2020).

Over the years, the hypothesis of immune and inflammatory dysregulation has been supported by research as a potential pathogenesis of schizophrenia (Miller and Goldsmith 2020; van Mierlo et al. 2020). For example, children of mothers who had elevated concentrations of CRP, IL-8, and IL-10 during pregnancy have been associated with a higher risk (J. Zhang et al. 2018). The first episode of psychosis (FEP) can be predicted by high IL-12 concentrations; also, IL-6 and TNF-α are associated with symptom severity and worsening negative symptoms in patients at risk of FEP (Stojanovic et al. 2014; Goldsmith et al. 2019; Park and Miller 2020).

For this reason, anti-inflammatory drugs have been investigated as potential augmentations for antipsychotics in the treatment of schizophrenia. However, the evidence for aspirin and minocycline is still inconclusive (Norbert Müller 2019; Mathes et al. 2019; Krynicki et al. 2021). Celecoxib, a selective COX-2 inhibitor, is another anti-inflammatory that exerts its effect through inhibiting prostaglandin and pro-inflammatory cytokines (B. Cohen and Preuss 2025).

The currently available RCTs provide conflicting evidence for the efficacy of Celecoxib in schizophrenia. The findings from clinical trials regarding efficacy have been inconsistent. While an earlier study reported no significant effect (Rapaport et al. 2005), recent larger RCTs showed clinical significance (Y. Zhang et al. 2021; Wang et al. 2024; Zarghami et al. 2024).

Previous meta-analyses, conducted in 2017 and 2019, do not support its overall efficacy as adjuvant treatment to anti-psychotic in schizophrenia (Zheng et al. 2017; Cho et al. 2019). Several limitations were identified in Zheng et al. (2017), which may undermine the reliability of the findings, for example, the inappropriate use of standardized mean difference. Although the inclusion of gray literature, such as conference abstracts, could reduce publication bias, but these often lack detailed methodology, particularly when these resources account for 50% of their sample size is another limitation (Hackenbroich et al. 2022; Paez 2017). Since 2019, additional RCTs have been published. By adding these studies, we aim to resolve the methodological flaws and limitations of the previous meta-analysis, enhancing statistical power and improving generalizability and reliability of findings. Given the substantial burden of schizophrenia on both patients and society—combined with the limited effectiveness of antipsychotics and notable side effects profile—our systematic review and meta-analysis aims to evaluate the efficacy and safety of celecoxib in schizophrenia to relieve this burden.

## Methods

We followed the preferred Reporting Items for Systematic review and Meta-Analysis (PRISMA statement) (Page et al. 2021) guidelines when reporting this study. This manuscript was conducted in adherence to the Cochrane Handbook of Systematic Reviews of Interventions (Cumpston et al. 2019). This study was registered on PROSPERO (CRD420251004700).

### Search strategy and data sources

Three independent authors searched PubMed, Scopus, Web of Science, and Cochrane for relevant studies in February 2025. For a sensitive search strategy, we used the MeSH keywords “Celecoxib “ in combination with “Schizophrenia “. The detailed search terms used for each database are illustrated in *Supplementary Table 1.*

### Selection Criteria

Studies satisfying the following inclusion criteria were included in the systematic review: (1) population: adult schizophrenia patients, (2) intervention: celecoxib alone or celecoxib combined with antipsychotics, (3) comparator: placebo alone or placebo with other antipsychotics, (4) outcome: studies reporting at least PANSS total score or PANSS sub-scales or other validated score that assess improvement such as BPRS or CGI scales, (5) Study design: randomized controlled trials (RCTs). We excluded articles that are (1) case reports/case series/observational studies/conference abstracts/thesis, (2) animal studies or in vitro studies, (3) reviews, book chapters, thesis, letters, overlapped datasets, and (4) non-English studies.

### Screening and study selection process

Three independent reviewers used EndNote for detecting duplicates, then Rayyan (Ouzzani et al. 2016) for semi-automated screening of the literature search results. Studies were screened in two phases: we screened the titles/abstracts for potential clinical studies in the first phase. In the second phase, we retrieved the full-text articles of the selected abstracts for further eligibility screening.

### Data extraction

For all included studies, data were extracted by two independent authors into an online data extraction sheet by independent authors, then the extracted data were compared to confirm accuracy. Extracted data included study characteristics, such as location, year, study design, population, sample size, intervention, dose, comparator, treatment duration, outcome measures, and conclusion of included studies. The second sheet included baseline characteristics of the included studies, such as age, male gender, marital status, educational level under diploma, duration of illnesses, and basal PANSS total, and the third outcome sheet was specifically designed to collect the outcomes of each study. Any disagreements in data extraction were resolved by consensus among the authors.

### Risk of bias assessment

Two independent authors assessed the risk of bias using the Online Cochrane Collaboration’s Risk of Bias 2 (ROB-2) tool, which evaluates five domains: D1 (bias arising from the randomization process), D2 (bias due to deviations from the intended intervention), D3 (bias due to missing outcome data), D4 (bias in the measurement of the outcome), and D5 (bias in the selection process of reported outcomes). The overall judgment for each domain was classified into one of three levels: low, moderate, or high risk of bias. Conflict among the authors was resolved through mutual agreement or by consulting the primary investigator.

### Grade assessment

We assessed the level of certainty of our evidence using the Grading of Recommendations, Assessment, Development, and Evaluation criteria (GRADE). The GRADE tool assesses the evidence based on the following domains: risk of bias (ROB), inconsistency, indirectness, imprecision, publication bias, and other factors and classifies it into four levels of certainty: very low, low, moderate, and high (Brignardello‑Petersen and Guyatt 2025).

### Measures of treatment effectiveness

The primary measure is the change in the Positive and Negative Syndrome Scale (PANSS) total score, which indicates symptom improvements. Other secondary measures include:

1- PANSS-negative subscale, PANSS-positive subscale, general psychopathology.

2- Adverse effects of Celecoxib.

### Data synthesis and statistical analysis

We analyzed data using RevMan software, version 5.4, R Studio. For continuous data, the mean difference (MD) between the two groups, along with its standard deviation (SD) and total number of patients, was pooled using the inverse variance method with a random effect model due to variability of treatment regimens. Heterogeneity was assessed using the chi-square test and I2 statistic; a *p* value from the chi-square test < 0.1 and I2 > 50% was considered indicative of significant heterogeneity. We conducted a leave-one-out sensitivity analysis to check if one study is responsible for the overall heterogeneity. Subgroup analysis according to treatment durations and meta-regression analyses were conducted to explore if any variables had potential effect on pooled analysis or contributed to the heterogeneity.

### Publication bias

We used the DOI plot to evaluate publication bias because there were less than ten included studies in the meta-analysis. Additionally, we calculated the Lafuente, Ferreira, and Kairalla (LFK) index to quantify the degree of publication bias. Values closer to 0 indicate minimal bias, while negative values beyond -1 and positive values beyond 1 suggest a likelihood of bias.

## Result

### Literature search results

**Fig. 1:**
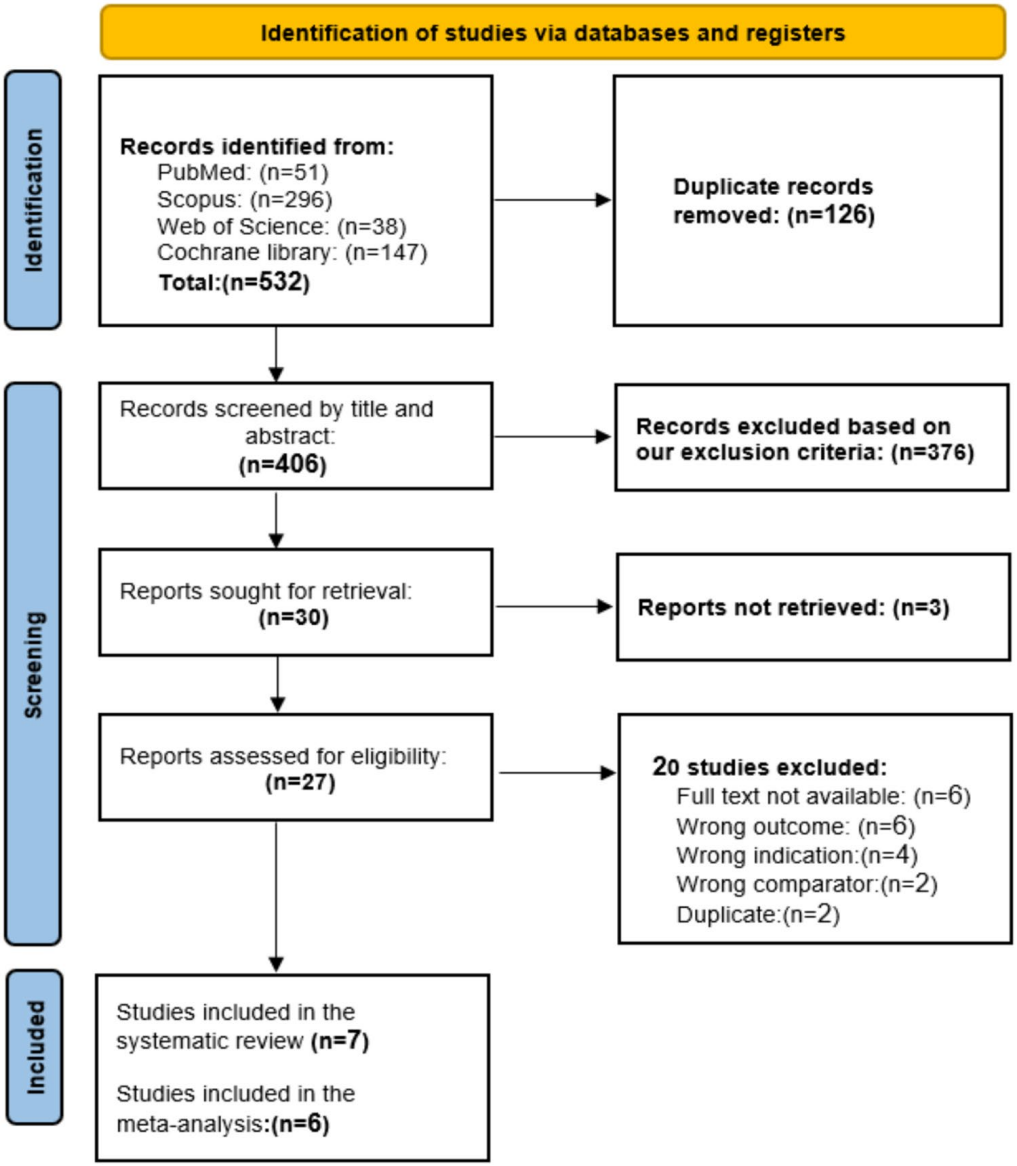
PRISMA flow diagram of included studies

We obtained 532 studies from the search, and EndNote identified 126 duplicates. After excluding irrelevant articles by screening using Rayaan, 27 articles were eligible for full-text screening. Of these, seven randomized controlled trials (RCTs) were included in this systematic review, and six of them were included in the meta-analysis. The PRISMA flow diagram of the study selection process is shown in (Fig. 1)**.**

### Characteristics of the included studies

Seven RCT studies comprising 438 patients with schizophrenia were included in this study. Two studies were conducted in Germany (Norbert Müller et al. 2002, 2010), two in Iran (Akhondzadeh et al. 2007; Zarghami et al. 2024), two in China (Y. Zhang et al. 2021; Wang et al. 2024), and one in the United States (Rapaport et al. 2005). The treatment duration ranged from five to twelve weeks. Only one study (Wang et al. 2024) et al. (2024) lasted 12 weeks. The dose on Celecoxib was 400 mg/day in most studies, except one study was 200 mg/day. Most of the investigated patients were on risperidone as anti-psychotic drugs, except for two studies: Müller et al., which used amisulpride, and Zarghami et al., which used haloperidol. Characteristics and a summary of the studies included are shown in Table 1 and Table 2.

**Table 1:** Summary of the included studies

Study ID	Location	Study design	Population	Sample size	Intervention	Dose	Comparator	Treatment duration	Outcome measures	Conclusion
Müller 2002	Germany	RCT	Patients with an acute exacerbation of schizophrenia. Diagnosis of schizophrenia according to the criteria of DSM-IV	50	Celecoxib plus risperidone	400 mg/ day	Placebo plus risperidone	5 weeks	PANSS total and sub-scales, Negative Symptoms, General Psychopathology, and extrapyramidal Symptoms	Adding celecoxib to risperidone improved schizophrenia symptoms faster and more effectively, especially between weeks 2 and 4. The benefits were not due to risperidone dosage differences, and celecoxib was well-tolerated. The study suggests that inflammation may play a role in schizophrenia
Rapaport 2005	United States	RCT	The study included medically stable schizophrenia patients who is continuously ill with a GAF score < 60, free from infections for 2 + weeks, and stabilized on olanzapine or risperidone for 12 + weeks	38	Celecoxib plus olanzapine or risperidone	400 mg/ day	Placebo plus olanzapine or risperidone	8 weeks	PANSS, CGI-S, CGI-I, Calgary Depression Rating Scale, Hamilton Anxiety Rating Scale, Barnes Akathisia Scale, Simpson-Angus Rating Scale, Abnormal Involuntary Movements Scale, and the Scale of Functioning	The trial investigated the effects of adjunctive celecoxib in schizophrenia patients stabilized on risperidone or olanzapine. There are no significant differences that were found between the celecoxib and placebo groups in symptom improvement, functioning, or side effects
Akhondzadeh 2007	Iran	RCT	The study included inpatients (aged 19–44) with chronic schizophrenia, meeting DSM-IV criteria and a PANSS score of at least 60. They had no recent infections, autoimmune diseases, or abnormal lab results and were off neuroleptics for at least one week	60	Celecoxib plus risperidone	400 mg/ day	Placebo plus risperidone	8 weeks	PANSS score from baseline, assessing schizophrenia symptoms. Extrapyramidal symptoms were evaluated using the ESRS, including parkinsonism, dystonia, and dyskinesia. Side effects were systematically recorded using a checklist throughout the study	This study evaluated the effects of adjunctive celecoxib on schizophrenia symptoms. Symptoms were assessed using PANSS, and side effects were monitored with the ESRS. Celecoxib group showed greater improvement in general psychopathology and total PANSS scores but had no significant impact on extrapyramidal symptoms or side effects
Müller 2010	Germany	RCT	Patients who is in Early stage with the diagnosis of schizophrenia (DSM-IV) or a schizophreniform disorder last for two years or less. The diagnoses were evaluated according to DSM-IV	50	Celecoxib plus amisulpride	400 mg/ day	Placebo plus amisulpride	6 weeks	PANSS (total, positive, negative, and global symptoms) and CGI for overall symptom severity. It also assessed extrapyramidal side effects, drop-out rates, and adverse events like the Bradycardia. Statistical analyses included mixed models and LOCF to compare outcomes	A significantly better outcome was observed in the patient group treated with amisulpride plus celecoxib compared to the group with amisulpride plus placebo. In addition, ratings by the CGI scale during therapy with amisulpride and celecoxib showed a significantly better result. A significantly superior therapeutic effect could be observed in the celecoxib group compared to placebo in the treatment of early-stage schizophrenia
Zhang 2021	China	RCT	The inclusion criteria were age 16–55 years, DSM-IV-TR diagnosis of schizophrenia in early stage, PANSS score > 60, disease duration < 6 months, anti-psychotic-naïve or treatment < 2 weeks, IQ ≥ 70, at least 6 years of education, and Has Chinese ethnicity	100	Celecoxib plus risperidone	200 mg/ day	Placebo plus risperidone	6 weeks	The circulating IDO levels measured at baseline and week 6. Secondary outcomes included serum levels of target cytokines (TNF-α, IL-1β, IFN-γ, IL-4, IL-6, IL-17) at baseline and week 6, as well as the total PANSS score and its subscale scores (Positive, Negative, and General) at both time points	Results showed that celecoxib significantly reduced IDO levels, improved negative symptoms, and decreased TNF-α and IL-1β levels compared to placebo. IDO reduction correlated with symptom improvement, suggesting a potential role of inflammation in schizophrenia
Wang 2024	China	RCT	Drug naïve first episode Patients had to establish a DSM-IV diagnosis of schizophrenia (SCZ) and meet the following criteria: be between 18 and 45 years old, experience an acute episode at admission, have symptoms lasting ≤ 60 months, and have not received prior anti-psychotic treatment	90	Celecoxib plus risperidone	400 mg/ day	Placebo plus risperidone	12 weeks	PANSS total and sub-scales, with response rates defined as a ≥ 50% reduction in total PANSS scores. Secondary outcomes assessed cognitive function (RBANS), side effects (TESS, SEPS, AIMS), and the influence of COX-2 genotypes on treatment response	In the clinical trial, adjunctive celecoxib (400 mg/day) significantly improved schizophrenia symptoms (PANSS scores) at weeks 10 and 12 compared to placebo but had no effect on cognitive function. The response rate was higher in the celecoxib group (66.7% vs. 26.3%). Celecoxib was well-tolerated, though it led to more weight gain. These findings suggest COX-2 inhibition as a potential therapeutic approach for schizophrenia, particularly in genetically predisposed individuals
Zarghami 2024	Iran	RCT	The study included chronic patients diagnosed with schizophrenia based on DSM-IV-TR criteria and required informed consent from both patients and their guardians	50	Celecoxib plus haloperidol	400 mg/ day	Placebo plus haloperidol	5 weeks	PANSS scores as the primary outcome. Secondary outcomes included drug side effects, drop-out rates, additional medication use, and safety concerns, including adverse events or deaths	The study found that celecoxib improved PANSS total scores more than placebo, particularly in positive symptoms and general psychopathology, but had no significant effect on negative symptoms. Celecoxib was well-tolerated, with no significant side effects

*RCT* Randomized controlled trial; diagnostic and statistical manual of mental disorders, *PANSS* Positive and negative syndrome scale, *GAF* Global assessment of functioning, *CGI*: Global impression scale, *IDO* Indoleamine 2,3-dioxygenase, *IFN* Interferon, *IL* Interleukin, *TNF* Tumor necrosis factor, *COX* Cyclooxygenase

**Table 2:** Baseline characteristics of the included studies

Study ID	Group name	Age (SD)	Male (%)	Duration of illnesses	Basal PANSS total
**Müller **2002	Celecoxib	35.9 (12.8)	N/A	N/A	71.7 (12.1)
	Placebo	35.5 (13.6)	N/A	N/A	75.5 (10.1)
**Rapaport **2005	Celecoxib	44.1 (9.2)	16 (88.9)	N/A	84.1 (11.4)
	Placebo	47.3 (11.4)	13 (76.5)	N/A	84.2 (12.9)
**Akhondzadeh **2007	Celecoxib	33.10 (7.29)	18 (60)	7.79 (5.87) years	92.8 (8.4)
	Placebo	34.30 (7.21)	17 (56.66)	7.98 (5.87) years	90.5 (9.4)
**Müller **2010	Celecoxib	26.2 (7.7)	14 (56)	16.0 (5.0) months	94.5 (16.2)
	Placebo	30.9 (8.1)	16 (64)	14.9 (4.6) months	95.9 (19.1)
**Zhang **2021	Celecoxib	30.46 (8.59)	34 (73.9)	N/A	90.17 (10.61)
	Placebo	27.85 (6.97)	29 (61.7)	N/A	94.00 (10.06)
**Wang **2024	Celecoxib	N/A	N/A	N/A	84.3 (18.2)
	Placebo	N/A	N/A	N/A	82.6 (20.2)
**Zarghami **2024	Celecoxib	37.71 (11.61)	20	N/A	86.96 (17.97)
	Placebo	36.57 (10.41)	19	N/A	80.29 (14.26)

All qualitative variables are represented as number (percentage), while quantitative variables are shown as mean (standard deviation)

*PANSS* Positive and Negative syndrome scale.

### Risk of bias assessment

**Fig. 2:**
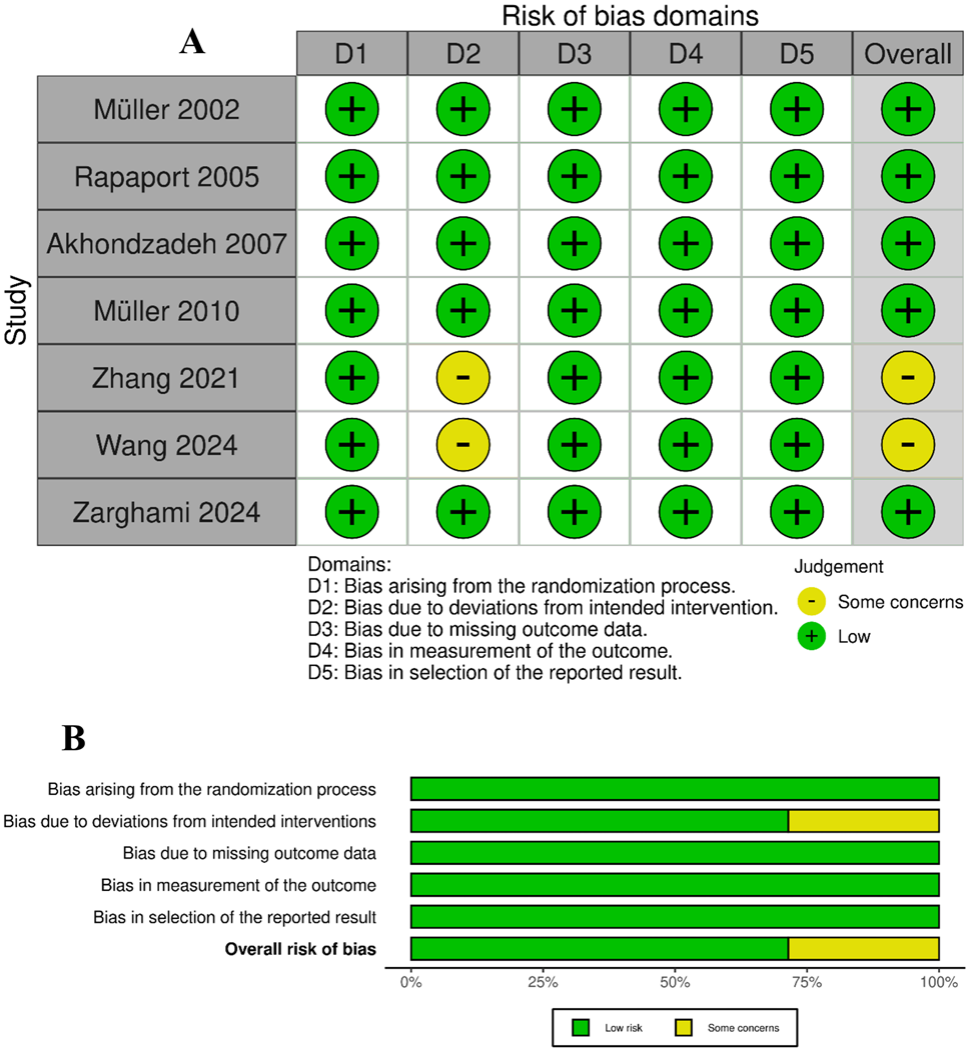
**A** Quality assessments according to risk of bias for each study. **B** Quality assessment according to risk of bias as percentage

A summary and a graph of the risk of bias in our included studies are shown in (Fig. 2). Results of the risk of bias assessment showed that the quality of the studies included ranged from low to moderate risk. Five studies (Norbert Müller et al. 2002; Akhondzadeh et al. 2007; Norbert Müller et al. 2010; Zarghami et al. 2024; Wang et al. 2024) showed a low risk of bias, but two studies (Rapaport et al. 2005; Y. Zhang et al. 2021) had some concern in domain 2.

### Meta-analysis for the primary outcome:

#### Celecoxib effect on PANSS total score:

**Fig. 3:**
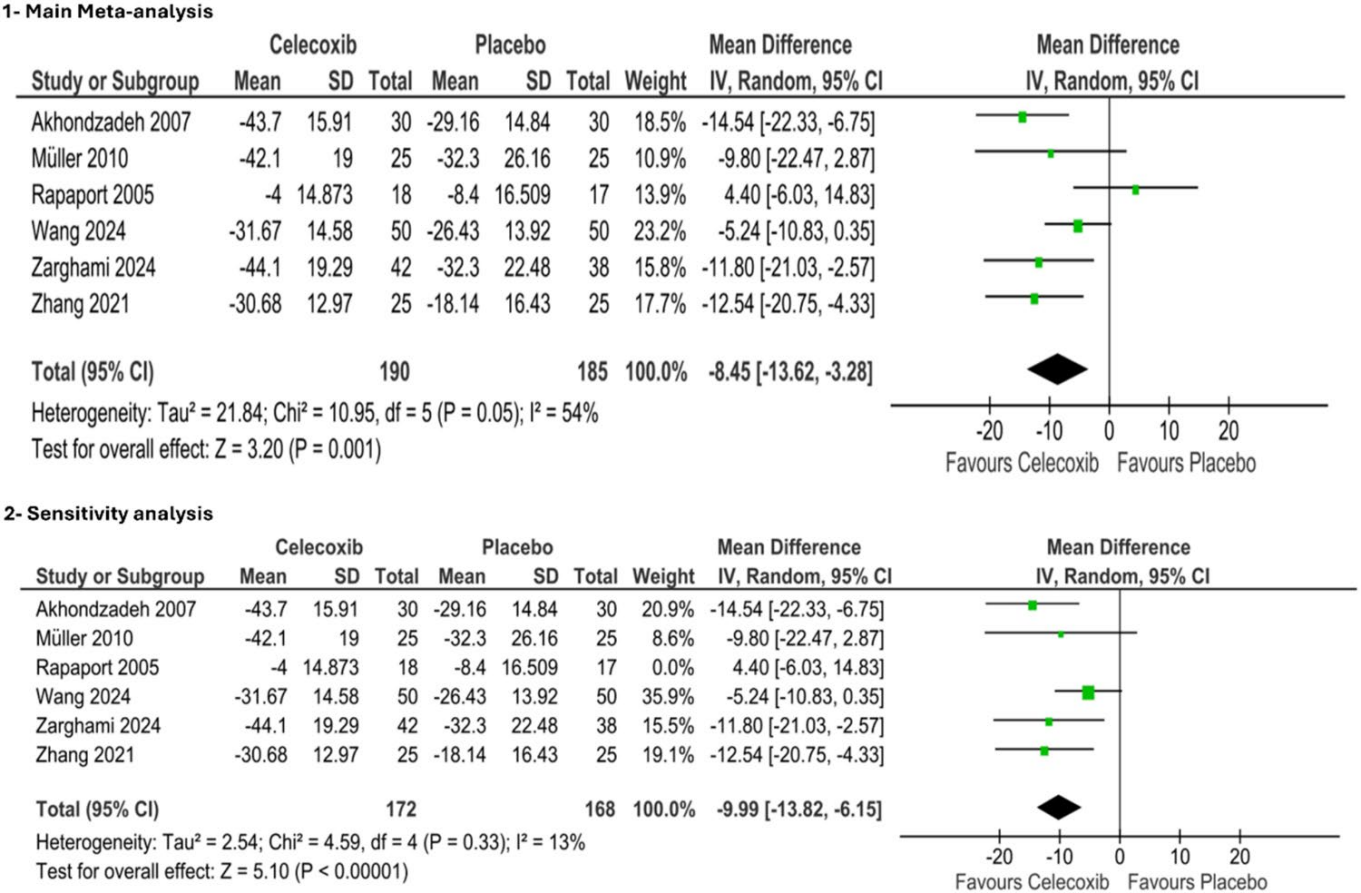
1-Forest plot of PANSS total score. 2-Leave-one-out sensitivity analysis: Sensitivity analysis for heterogeneity. *IV* Inverse variance, *CI* Confidence interval

Six studies only reported the change in PANSS total score with a sample of 375 patients. The pooled effect statistically significantly favored celecoxib over the control group (MD = − 8.45, 95% CI [− 13.62, -3.28], *p* value = 0.001). The pooled studies were heterogeneous (chi-square *p* = 0.05, I2 = 54%) as shown in (Fig. 3).

### Sensitivity analysis for PANSS total score

We conducted a leave-one-out sensitivity analysis by excluding one study at a time to investigate if a particular study accounted for the heterogeneity. The heterogeneity was resolved by excluding Rapaport et al. (2005). The overall effect still favored celecoxib over placebo (MD = − 9.99, 95% CI [− 13.82, − 6.15], *P* < 0.00001). The pooled studies were homogenous (chi-square *p* = 0.33, I2 = 13%), as shown in (Fig. 3).

### Subgroup analysis for PANSS total

We conducted subgroup analysis to investigate the efficacy of the drug according to duration. We found that the drug was significant in 6 weeks (MD = − 7.29, 95% CI [− 12.11, − 2.47], *p* = 0.003). Also, Wang et al. (2024) showed significant efficacy in 10 weeks (MD = − 9.4, 95% CI [− 18.62, − 0.18], *p* = 0.05) and in 12 weeks (MD = − 7.66, 95% CI [− 11.44, − 3.88], *p* < 0.0001), but the drug was not significant in 8 weeks (MD = − 5.51, 95% CI [− 16.35, 5.32], *p* = 0.32). Furthermore, no significant differences were observed among the subgroups (*p* = 0.80) as shown in (**Supplementary Fig. 1**).

### Meta-regression for PANSS total score

Meta-regression analyses were conducted to explore if any variables had potential effect on pooled analysis or contributed to the heterogeneity. One variable is the chlorpromazine-equivalent dose of antipsychotics used as adjunct to celecoxib (Leucht et al. 2009, 2015). For chlorpromazine-equivalent dose, the coefficient was –0.011 (*p* = 0.09), indicating that different antipsychotics used had no significant effect on the efficacy of celecoxib. However, this variable reduced the heterogeneity, with τ2 decreasing from 21.80 to 17.65 and I2 from 54 to 48%, accounting for approximately 21.75% of the observed heterogeneity. The disease duration coefficient was 0.029 (*p* = 0.3), but the heterogeneity increased after including this variable (τ2 increased to 33.90, I2 = 59.8%), providing no explanation for the heterogeneity. Regarding different treatment duration, the coefficient was − 0.24 (*p* = 0.80), indicating no significant relation between the celecoxib duration and the effect on PANSS score. Moreover, heterogeneity increased post-regression (τ2 = 33.90, I2 = 64.76%). The details of the meta-regression analysis are presented in Table 3.

**Table 3:** Results of meta-regression analyses examining the effect of potential variables on the efficacy of celecoxib

		*p *value	Heterogeneity before meta-regression (τ2, I2)	Heterogeneity after meta-regression (τ2, I2)	Proportion of heterogeneity explained by the model (R2 analog)
Chlorpromazine-equivalent dose of adjunct anti-psychotic (mg/day)	− 0.011	0.09	τ2 = 21.8, I2 = 54%	τ2 = 17.65, I2 = 48%	21.75%
Disease duration (months)	0.029	0.3		τ2 = 33.9, I2 = 59.8%	26.08%
Treatment duration (weeks)	− 0.24	0.8		τ2 = 33.9, I2 =	

### Publication bias for PANSS total score

**Fig. 4:**
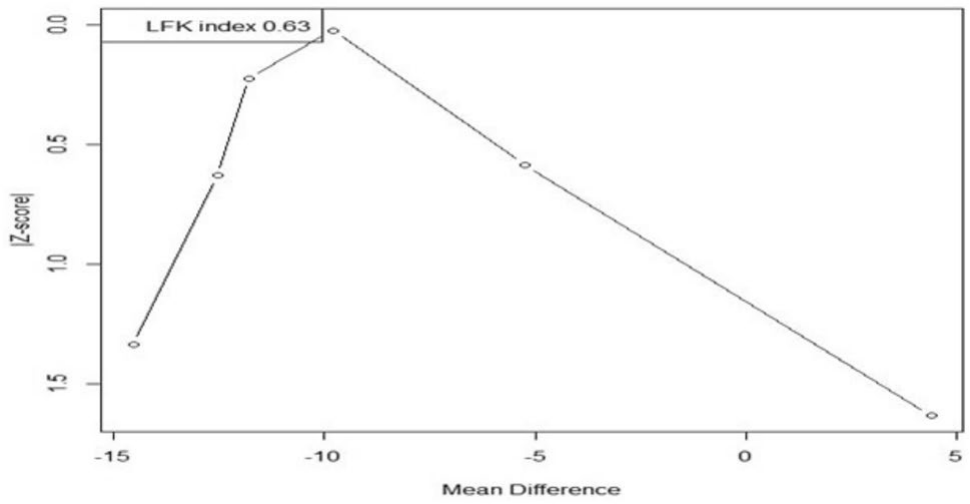
DOI plot for publication bias. LFK index: Luis Furuya-Kanamori

Visual inspection of DOI plot did not indicate publication bias, which was further confirmed by the calculated LFK index of -0.63 that indicated no asymmetry as shown in (Fig. 4).

### Meta-analysis for the Secondary outcomes

**Fig. 5:**
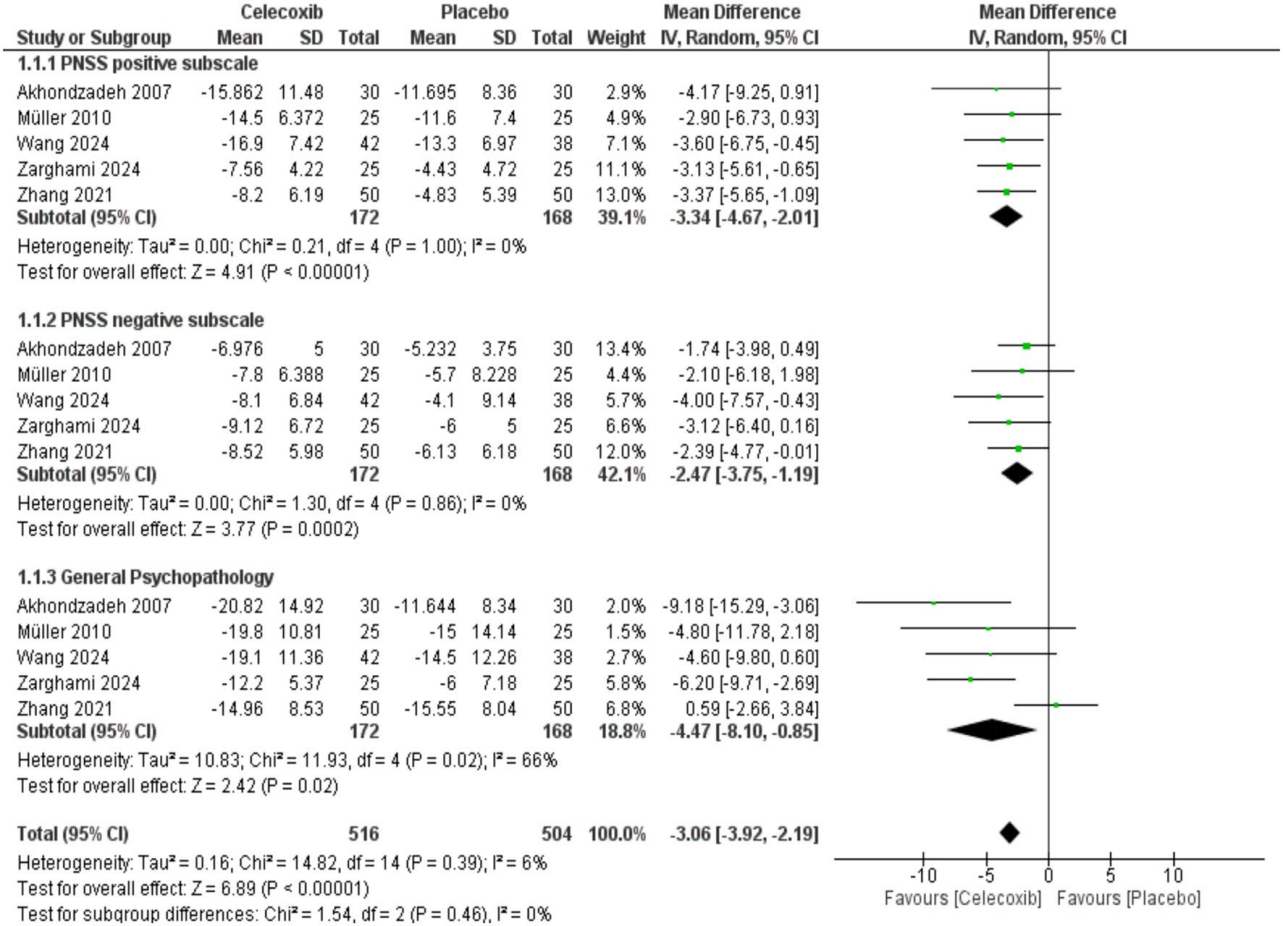
Forest plot of **A**: PANSS-positive subscale, **B**: PANSS-negative subscale, **C**: General psychopathology. *IV* Inverse variance, *CI* Confidence interval

Five studies reported PANSS subscale outcomes. Celecoxib was statistically superior to placebo in PANSS-positive subscale (MD = − 3.34, 95% CI [− 4.67, − 2.02], *P* < 0.00001, I2 = 0%), PANSS-negative sub-scales (MD = − 2.47, 95% CI [− 3.75, − 1.19], *P* = 0.00002, I2 = 0), and general psychopathology (MD = − 4.47, 95% CI [8.10, − 0.85], *p* = 0.02, I2 = 66) as shown in (Fig. 5). The pooled studies were homogenous in PANSS-positive and -negative sub-scales, but a significant heterogeneity was noticed in general psychopathology (chi-square *p* = 0.02, I2 = 66%), which was resolved by excluding Zhang et al. (2021) in the leave-one-out sensitivity analysis (chi-square *p* = 0.7, I2 = 0%) as shown in **(Supplementary Fig. 2)**.

### Adverse effect

Most of the studies reported that there was no significant difference between the Celecoxib add-on therapy group and the placebo group in adverse events and side effects. However, they did not report the numerical data, limiting the ability to conduct meta-analysis. Müller et al. (2002) reported that there was one patient drop-out in the placebo and risperidone group due to gastrointestinal problems, and the drop-out patients in the celecoxib group were due to risperidone side effects (edema, changes in the ECG). In Müller et al. (2010), one patient had to be withdrawn due to asymptomatic bradycardia, a well-known side effect of amisulpride; otherwise, there were no serious side effects noticed.

### GRADE assessment

Based on GRADE assessment, the quality of evidence on the change of PANSS total score (MD = − 8.45, 95% CI [− 13.62, -3.28], I^2^ = 54%) was evaluated as low. The quality of evidence was limited by imprecision and indirectness domains. The details of the GRADE assessments are provided in the **Supplementary Table 2.**

## Discussion

Schizophrenia is a lifelong, debilitating disorder, and many researchers support that immune and inflammatory dysregulation plays a key role in its pathogenesis (Bian et al. 2008; Sugino et al. 2009). Our meta-analysis aims to investigate the role of celecoxib in schizophrenia, as celecoxib had already shown a significant improvement as adjunct therapy in other psychiatric disorders by targeting the inflammatory pathway (Na et al. 2014; Faridhosseini et al. 2014; Bavaresco et al. 2019).

Our study showed that the celecoxib was superior to placebo in improving the PANSS total score with a significant reduction in symptoms of schizophrenia; the analysis came with heterogeneity, which was resolved by leave-one-out sensitivity analysis when excluding Rapaport et al. (2005). This may be explained by this study, which reported that Celecoxib is not significant as an adjunct drug in the management of schizophrenia, had the smallest sample size among the included studies, making it more susceptible to random variability and reducing its statistical power. Additionally, Rapaport et al. (2005) differed demographically from other studies, with the oldest sample size (celecoxib: 44.1 years, placebo: 47 years), whereas the age range in other studies was 26.2 years to 37.7 years. Moreover, it had the highest proportion of male participants (88.9%); these factors could contribute to the variation in treatment response of this study compared to other studies, leading to the heterogeneity. Also, the difference of clinical presentation of the patient may contribute to heterogeneity as Rapaport et al. (2005) were on continuously ill patients while Müller et al. (2010) and Akhondzadeh et al. (2007) were on early staged patients, Zhang et al (2021) and Zarghami et al. (2024) were on chronic patients and Wang et al. (2024) were on first episode drug-naïve patients.

We conducted a subgroup analysis according to duration. The analysis of four RCTs at six weeks showed that the celecoxib was superior to placebo (Akhondzadeh et al. 2007; Norbert Müller et al. 2010; Y. Zhang et al. 2021; Wang et al. 2024), but at eight weeks, three RCTs showed no significant difference between the two groups (Rapaport et al. 2005; Akhondzadeh et al. 2007; Wang et al. 2024). Also, one study (Wang et al. 2024) found that the drug is significant at 10 weeks and 12 weeks. The analysis was non-conclusive due to the small number of studies, so we cannot determine exactly the correlation between the efficacy and the duration, but 6 weeks is the most reliable duration.

We conducted meta-analysis regression to explore if any variable contributed on heterogeneity, we found that none of disease duration or duration of treatment variable explain the heterogeneity and for chlorpromazine-equivalent dose variable, the coefficient was –0.011 (*p* = 0.09), indicating that different antipsychotics used had no significant effect on the efficacy of celecoxib. However, the variable reduced approximately 21.75% of observed heterogeneity. This difference of antipsychotics may explain the source of heterogeneity as all trials used antipsychotics which are superior to haloperidol except for Zarghami et al (2024) who used haloperidol (15–30 mg). Also, Rapaport et al (2005) used smaller dose (3 mg) of risperidone and clozapine. Müller et al (2010) continued to show no effect although using amisulpride which is the second-best anti-psychotic after clozapine. In addition to that, none of our studied used aripiprazole, which documented anti-inflammatory effects, so we recommend future studies to may use Celecoxib as an adjunct to aripiprazole to explore the maximum anti-inflammatory effect (Loucera‑Muñecas et al. 2024).

Across five included studies in our secondary analysis, the drug showed significant improvement in all secondary outcomes: PANSS-positive subscale, PNSS-negative subscale, and general psychopathology. In general psychopathology analysis, there was significant heterogeneity resolved by excluding Zhang et al. (2021) as this study showed no significant difference between celecoxib and placebo groups.

Cox-2 inhibitor is believed to have a beneficial effect in the treatment of cognitive impairment through its role in reducing inflammation as the previous study has proved its role in diseases that associated with cognitive impairment such as Alzheimer disease (Jordan et al. 2020). Also, a study conducted on schizophrenia patients found that celecoxib adjuvant to risperidone had positive effects in the treatment of cognitive impairment (Norbert Müller et al. 2005). In addition, animal study has shown that celecoxib can improve the cognitive dysfunction of ischemic rats by inhibiting inflammation because it can inhibit COX-2 (Gou et al. 2021). However, Wang et al. (2024) have found that celecoxib did not have a better effect on cognitive impairment in patients with DNFE (drug-naïve first episode) SCZ compared to placebo as measured by RBANS (Repeatable Battery for the Assessment of Neuropsychological Status). This may be due to the insufficient sensitivity of the cognitive function test they used to identify subtle changes in this function. However, Celecoxib showed a good safety profile as no serious side effects had been noticed in all studies, but as we know, schizophrenia is a long-term chronic disability disease that may be associated with a risk of comorbidities, especially in elderly patients with chronic schizophrenia, as they have a higher risk of congestive heart failure, hypothyroidism, metabolic syndrome, and chronic obstructive pulmonary disease (Hagi et al. 2021; C. I. Cohen et al. 2008). Also, long-term treatment with anti-psychotic is associated with side effects as weight gain, movement disorder, and sedation (Gaebel et al. 2020; Leucht et al. 2012).

Moreover, NSAIDs (non-steroidal anti-inflammatory drugs) have a higher risk for renal toxicity, which occurs in 5% of patients who take these agents, whether they are non-selective or selective COX-2 inhibitors (Cyclooxygenase-2 inhibitor). Celecoxib, for example, has been shown to cause renal dysfunction, which is at a higher risk in elderly patients than younger patients (Morales and Mucksavage 2002; Lucas et al. 2019; Bindu et al. 2020). Also, NSAIDs have a cerebrovascular risk through many mechanisms as NSAIDs have an effect on increasing blood pressure since the COX inhibitor effect and inhibition of prostaglandin (PG) synthesis can induce vasoconstriction and volume expansion due to impaired sodium excretion. In addition, the prothrombotic effect of the COX-2 inhibitor through endothelium damage can increase the risk of cerebrovascular disease, which also contributes to cardiovascular risks and gastrointestinal bleeding (Hersh et al. 2007; Lapi et al. 2016; Fanelli et al. 2017). According to this, celecoxib may have a risk in elderly patients with schizophrenia, especially those who have comorbidities.

Our findings can be attributed to the pathogenesis of the immune and inflammatory dysregulation in schizophrenia. Chronically activated macrophages and T lymphocytes with excessive IL-2 and other cytokines have been found as a cause of schizophrenia (Licinio et al. 1993; Kronfol and Remick 2000; Cazzullo et al. 2001). As it is reported that CSF levels of IL-2 and sIL-2R, soluble IL-6 receptors that are a functional part of the IL-6 system, and IL-10 are high in schizophrenic patients. The high levels of cytokines in the CNS compartment may be accompanied by increased COX-2 expressions (McAllister et al. 1995; N Müller et al. 1997; van Kammen, McAllister, and Kelley 1997). According to this, Müller et al. (2002) hypothesize that celecoxib down-regulates the cytokine-induced CNS COX-2 activation.

Celecoxib, an anti-inflammatory COX-2 inhibitor, regulates pro-inflammatory cytokine secretion by inhibiting the synthesis of pro-inflammatory prostaglandins and rebalancing T lymphocyte response with a neuro-protection mechanism (Riedel et al. 2005).

### Previous studies

The latest meta-analysis published by Cho et al. (2019) investigated adjunctive use of anti-inflammatory drug for schizophrenia (Cho et al. 2019). They included four RCTs encompassing 195 patients and reported no significant difference between add-on celecoxib and standard anti-psychotic treatments in terms of PANSS total and negative scores.

Another meta-analysis was conducted in 2017, with a sample size of 626 patients [18]. Notably, more than half of the sample size (330) was included from two conference abstracts. These abstracts, which we were unable to access during our review, were not peer-reviewed for full-text publications, raising concerns about reporting quality and long-term accessibility. In contrast, our meta-analysis included three newly published RCTs and excluded these abstracts to reduce potential bias. Additionally, both abstracts were published by the same author (Muller) who published two full-text studies (Muller. 2002 and Muller. 2010) included in their meta-analysis, introducing a possible risk of overlapping sample. Another methodological difference is the use of standardized mean difference (SMD) in their analysis. We pooled the effect using mean difference (MD) to allow for more meaningful clinical interpretation. Taking into consideration these methodological differences along with our updated data may help explain the discrepancies between our study findings compared to prior research.

### Strength points and limitations

Our study is the most up-to-date meta-analysis on the use of Celecoxib in the management of schizophrenia, encompassing data from seven RCTs. Our meta-analysis improves the previous meta-analysis by adding three new RCTs and implementing methodologically rigorous approaches to ensure greater accuracy, reliability, and clinical relevance. A key strength of our study is the robustness of our findings; results from leave-one-out sensitivity analyses consistently aligned with the pooled analysis of the PANSS total score, demonstrating a significant advantage of celecoxib over placebo. Additionally, most studies yielded low risk of bias according to Cochrane ROB-2 tool, indicating high methodological quality. Furthermore, the geographic diversity of the included studies further supports the external validity of our results, with data spanning Western, Eastern, and Middle Eastern countries.

However, our study was not without limitations. The limited number of RCTs and the small sample size of 438 patients are significant limitations, in addition to the lack of non-English study and not using gray literatures. Also, the antipsychotics used were not the same. The clinical presentation and the duration of the trials were different, which could explain the heterogeneity found in some of the analyses. Heterogeneity and small sample size may limit the generalizability of our results. Long-term safety and adverse effects of celecoxib are not well-explained. Only Wang et al. (2024) investigated the efficacy of the drug as an adjunct in a long-term period of 10 and 12 weeks. The dose of the drug was 400 mg/day in most of the studies. Only in Zhang et al. (2021), it was 200 mg/day. Also, according to grade assessment, the quality of evidence on the change of PANSS total score was evaluated as low as it was limited by imprecision and indirectness domains.

### Significance of the findings and future research

Our study provides a contribution to the literature about the efficacy and safety of celecoxib in schizophrenia as our study findings support the role of celecoxib as an adjunct therapy in schizophrenia. These results are important in clinical practice as schizophrenia is a challenging disease to treat. Although our pooled effect (MD = –8.45, 95% CI [–13.62, –3.28]) was statistically significant, it did not exceed the minimum clinically important difference (MCID) for the PANSS total score (14.02 to 31.5) as reported by Si et al. (2021) (Si et al. 2021). However, this MCID was established based on a population with acutely exacerbated schizophrenia, where our meta-analysis included a more heterogeneous sample ranged from acute to chronic patients. While celecoxib yielded a statistically significant reduction, its impact may not be sufficient to produce a meaningful clinical improvement.

We recommend further research be conducted with larger sample sizes with standardized treatment duration and consistent anti-psychotic co-administration to confirm long-term efficacy and safety of celecoxib in schizophrenia. Additionally, studies should explore different response across patients’ subgroups, particularly considering the demographic and inflammatory biomarkers profile to identify individuals who may benefit the most from celecoxib as an adjunct in schizophrenia. Also, we suggest investigating short-term adjunctive use (e.g., during acute inpatient care) in real-world settings.

## Conclusion

In conclusion, our meta-analysis shows that celecoxib, when combined with antipsychotics, significantly reduces the total PANSS score in schizophrenia. However, our analysis is limited by small sample sizes and varying treatment durations, which warrants cautious interpretation of the findings. While these results indicate that celecoxib could be a promising adjunct to antipsychotics in the management of schizophrenia, further research involving larger, standardized trials is needed to confirm these findings and explore variations in response according to inflammatory and demographic biomarkers.

## Supplementary Information

Below is the link to the electronic supplementary material.

Supplementary file1 (DOCX 633 KB)

## Data Availability

Data and material are available within the manuscript.
