# Supplementary material for: Efficacy and safety of celecoxib in schizophrenia: a systematic review and meta-analysis of randomized controlled trials
Source: Inflammopharmacology. 2025 Aug 20;33(9):5049–65. doi: 10.1007/s10787-025-01908-6 (PMC12443880; doi:10.1007/s10787-025-01908-6)
Supplement: Supplementary file 1 — Supplementary file1 (DOCX 633 KB) [file 10787_2025_1908_MOESM1_ESM.docx]

| **Appendix A**: databases were used to search for articles related to the following key words: | | |
| --- | --- | --- |
| Number | Search strategy | Database |
| 51 | (celecoxib OR celebrex OR "COX-2 inhibitor") AND ("schizophrenic spectrum disorder" OR "chronic schizophrenia" OR "schizophrenic disorders" OR schizophrenia) | PubMed |
| 296 | ( TITLE-ABS-KEY ( ( celecoxib OR celebrex OR "COX-2 inhibitor" ) ) AND TITLE-ABS-KEY ( ( "schizophrenic spectrum disorder" OR "chronic schizophrenia" OR "schizophrenic disorders" OR schizophrenia ) ) ) | Scopus |
| 147 | ALL=((celecoxib OR celebre OR "COX-2 inhibitor") ) AND ALL=(("schizophrenic spectrum disorder" OR "chronic schizophrenia" OR "schizophrenic disorders" OR schizophrenia)) | Web of science |
| 38 | (celecoxib OR celebrex OR "COX-2 inhibitor") AND ("schizophrenic spectrum disorder" OR "chronic schizophrenia" OR "schizophrenic disorders" OR schizophrenia) | Cochrane library |
| 532 | The total from all four databases: | |
| 126 | Number of duplicates detected by (End Note): | |
| 406 | Number after deleting duplicates: | |

**Supplementary Table 1:** Search strategies and results for each database.


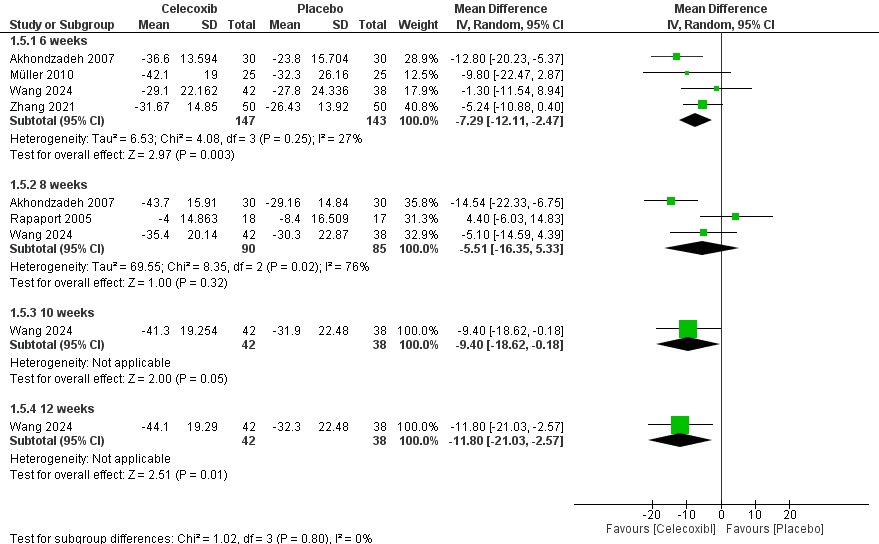
 **Figure 1:** Sub-group analysis of PANSS total score according to treatment duration. IV: Inverse variance; CI: Confidence interval


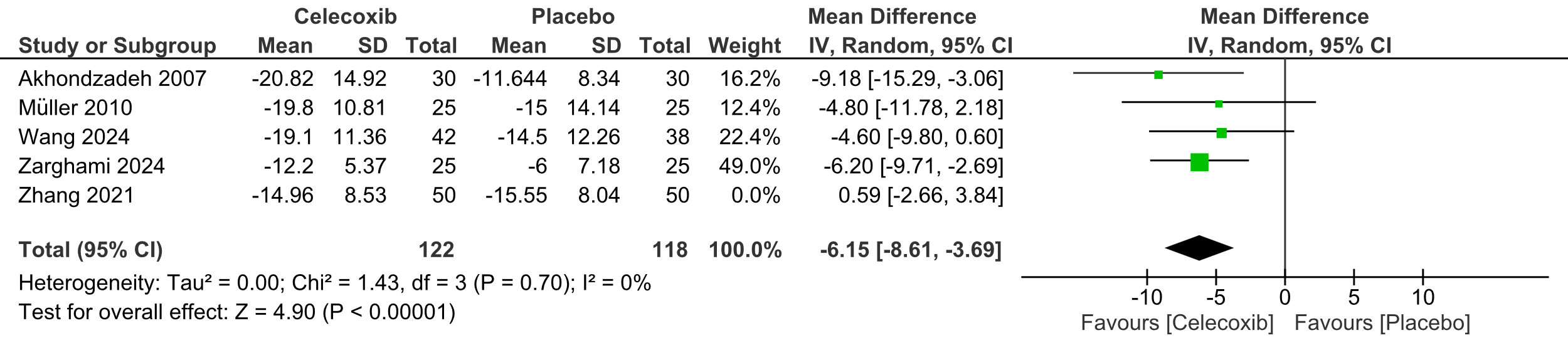


**Figure 2:** leave-one-out sensitivity analysis for general psychopathology heterogenicity. IV: Inverse variance; CI: Confidence interval

|  | **Risk of Bias** | **Inconsistencies** | **Indirectness** | **Imprecision** | **Publication bias** | **Others** | **Final assessment** |
| --- | --- | --- | --- | --- | --- | --- | --- |
| **PANASS total score** | No | Downgrade by one level * | Downgrade by one level ** | No | N/A | No | Low |

**Table 2.** GRADE assessment of the certainty in evidence in the primary outcome. *Note*: N/A = Not Applicable because of the small number of studies included (Egger et al.).

- * Owing to the significant heterogeneity in the effect estimate (I² = 54%).
- ** Owing to variations in the stage of schizophrenia (early vs chronic) and variability in the antipsychotic agents used as adjuncts to celecoxib
